# Exploring the Molecular Basis for Selective Binding of Homoserine Dehydrogenase from *Mycobacterium leprae TN* toward Inhibitors: A Virtual Screening Study

**DOI:** 10.3390/ijms15021826

**Published:** 2014-01-24

**Authors:** Dongling Zhan, Dongmei Wang, Weihong Min, Weiwei Han

**Affiliations:** 1 Key Laboratory for Molecular Enzymology and Engineering of Ministry of Education, Jilin University, Changchun 130023, China; E-Mail: gykfamily@163.com (D.W.); 2 College of Food Science and Engineering, Jilin Agricultural University, Changchun 130118, China; E-Mail: zdlgale@126.com (D.Z.)

**Keywords:** homology modeling, molecular dynamic, virtual screening, docking

## Abstract

Homoserine dehydrogenase (HSD) from *Mycobacterium leprae TN* is an antifungal target for antifungal properties including efficacy against the human pathogen. The 3D structure of HSD has been firmly established by homology modeling methods. Using the template, homoserine dehydrogenase from *Thiobacillus denitrificans* (PDB Id 3MTJ), a sequence identity of 40% was found and molecular dynamics simulation was used to optimize a reliable structure. The substrate and co-factor-binding regions in HSD were identified. In order to determine the important residues of the substrate (l-aspartate semialdehyde (l-ASA)) binding, the ASA was docked to the protein; Thr163, Asp198, and Glu192 may be important because they form a hydrogen bond with HSD through AutoDock 4.2 software. After use of a virtual screening technique of HSD, the four top-scoring docking hits all seemed to cation–π ion pair with the key recognition residue Lys107, and Lys207. These ligands therefore seemed to be new chemotypes for HSD. Our results may be helpful for further experimental investigations.

## 1. Introduction

Fungal infections have escalated dramatically in recent years [1]. This escalation can be attributed to three factors. Firstly, the number of immunocompromised individuals such as AIDS patients, organ transplant recipients and those undergoing chemotherapy has steadily increased [2]. Secondly, drug resistance against many existing antifungal therapies has arisen in fungi. Finally, previously benign fungi have now become the cause of systemic mycoses, thus increasing the list of pathogenic fungi. As a consequence of the developing threat of fungal infections, attention has been focused on the identification and exploration of novel fungal targets.

Homoserine dehydrogenase (HSD) was identified as an antifungal target when it was shown that a natural amino acid second metabolite (*S*)-2-amino-4-oxo-5-hydroxypentanoic acid, had antifungal properties including efficacy against the human pathogen *Candida Albicans* [3,4]. The target for this natural compound proved to be HSD, an enzyme that is required for the biosynthesis of the three essential amino acids, methionine, isoleucine and threonine [4,5]. HSD is found within the animal kingdom, making HSD an ideal target for the structure-based design of antimycotic drugs.

Homoserine dehydrogenase belongs to the expansive and diverse class of oxidoreductases. HSD shares certain similarities with other dehydrogenases, such as malate, lactate and glyceraldehyde 3-phosphate dehydrogenase [6,7]. For instance, the cofactor NAD(P)H binds to a nucleotide-binding domain that conforms to the Rossmann fold [8,9]. However, HSD displays several crucial differences from all other dehydrogenases. First, the overall fold of the catalytic region is unique among all known protein structures; Second, residues that have been implicated in catalysis in other oxidoreductase enzymes are not present in the active site of HSD. As such, HSD represents a novel enzyme within the oxidoreductase class [10–13].

Until now, there were eight structures determined by experiment [4,14,15]. The rate of the 3D structure of HSD determined is lower than that of the need of development of antimycotic drugs. Thus, a homology model was used to build a 3D structure of HSD. The present study is aimed at elucidating the 3D structural features of homoserine dehydrogenase (HSD) from *Mycobacterium leprae TN* and selective prediction of interaction sites for substrates and inhibitors. In this study, we report that the 3D model of HSD was derived using comparative modeling analysis [16,17] and that the generated 3D models would give insight into the influence of various interactive fields on the activity and thus, can help in designing and forecasting the translation inhibition activity of novel molecules. Further, refinement of the generated 3D model was done by subjecting it to molecular dynamics (MD) simulations. Molecular docking studies were also performed to analyze the interactions amongst HSD and its ligands, which are found to be helpful in the design of a novel antimycotic drug.

## 2. Results and Discussion

### 2.1. Sequence Alignments and Molecular Modeling

**Figure 1.**
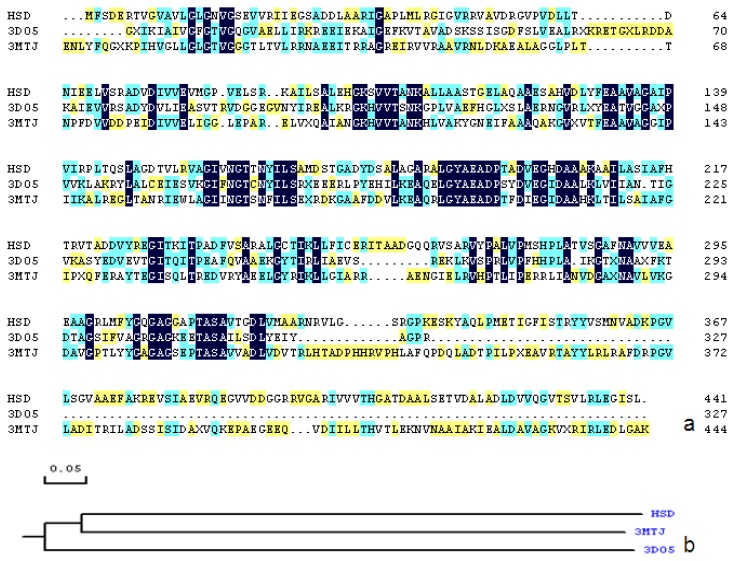
(**a**) Sequence alignment of HSD homologs. 3MTJ (40%); 3DO5 (41%); (**b**) Phylogenetic tree of HSD, 3MTJ, and 3DO5.

Among the BLASTp results, the structure was selected as templates: homoserine dehydrogenase from *Thiobacillus denitrificans* (PDB code 3MJT). The sequence identities between HSD and templates 3MJT was 40%. It well known that above 50% sequence identity, models tend to be reliable, with only minor errors in side chain packing and rotameric state. In the 30%–50% identity range, errors can be more severe and are often located in loops. Below 30% identity, serious errors occur, sometimes resulting in the basic fold being mis-predicted [18]. Thus, 40% identity is sufficient homology to construct a believable model [19]. The sequence alignment performed using the MolsoftICM for homology modeling is shown in Figure 1a. Although the sequence identities between HSD and templates: Putative Homoserine Dehydrogenase (NP_069768.1) from *Archaeoglobus Fulgidus* (PDB ID 3DO5) (41%) is higher than that of HSD and 3MJT’s, 3MJT was chosen as template. The reasons are as follows: firstly, 3MJT contains 496 residues, and 3DO5 contains 327 residues. As a template, the length of 3MJT is more appropriate than that of 3DO5’s. Secondly, phylogenetic analysis (seen from Figure 1b) showed that 3MJT and HSD are the same subfamily, and thus their spatial structure should be more similar. The most significant step in homology modeling process is to obtain the correct sequence alignment of the target sequence with the homologues, and it reveals that the residues involved in binding of substrate in templates (Lys217 (Proton donor)), Arg117 (NADH binding) and Glu196 (substrate binding site) were conserved (the corresponding residue: Lys207, Arg107 and Glu192) in HSD.

The coordinates of the crystal structures of homoserine dehydrogenase from *Thiobacillus denitrificans* (PDB code 3MJT) was used as templates to build the structure of HSD. The 3D model of the HSD was built by Swiss model [16,17]. Further, refinement was performed in order to obtain the best conformation of the developed model of HSD. Analysis of 20 ns dynamics shows that the HSD structure is stable and indicated that the system is stable.

**Figure 2.**
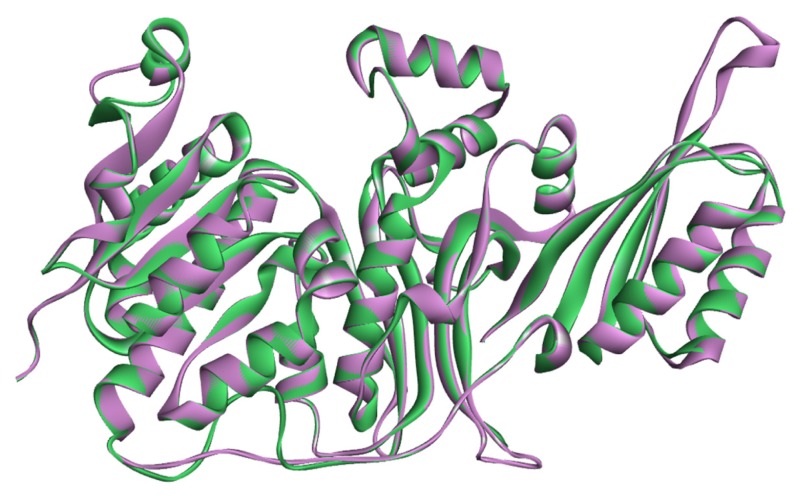
The superposition of the average structure of the HSD (pink) with the initial model (green).

The superposition of the average structure of the HSD with the initial model Figure 2. Figure 2 does not show major structure conformational changes in comparison to the initial model, which is consistent with the relatively low RMSD values. We selected the average structure of the HSD through the further study.

### 2.2. Validation of Homology Model

**Figure 3.**
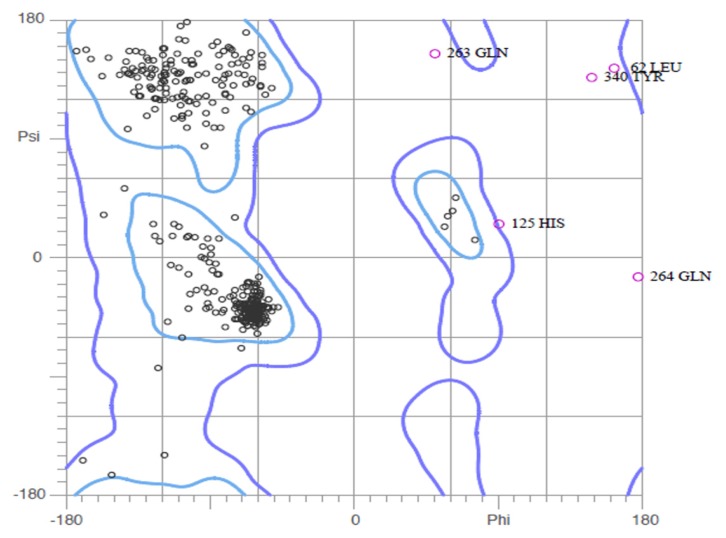
Ramachandran plot.

The first validation was carried out using Ramachandran plot calculations computed with Molprobity program by checking the detailed residue-by-residue stereo-chemical quality of a protein structure [20–22]. The results are shown in Figure 3. Altogether, 95.0% of all residues were in favored regions, and 98.4% of all residues were in allowed regions. In comparison with the homology model, the template, 3MJT, had a similar Ramachandran plot 98.35% in the allowed regions.

Seen from Figure 3, it can be concluded that Glu263, Leu62, Tyr340, and Gln264 are in the disallowed regions. So we fixed them by hand. ERRAT is a so-called “overall quality factor” for nonbonded atomic interactions, and higher scores mean higher quality [23]. The normally accepted range is >50 for a high quality model [23]. In the current case, the ERRAT score for HSD model is 87.50, well within the range of a high quality model, in the mean time the ERRAT score for the templates 3MTJ is 96.95. Thus, the above analysis suggests that the backbone conformation and non-bonded interactions of HSD homology model is all reasonable within a normal range. The final evaluation of the built HSD structure was checked by Verify 3D [24,25].

**Figure 4.**
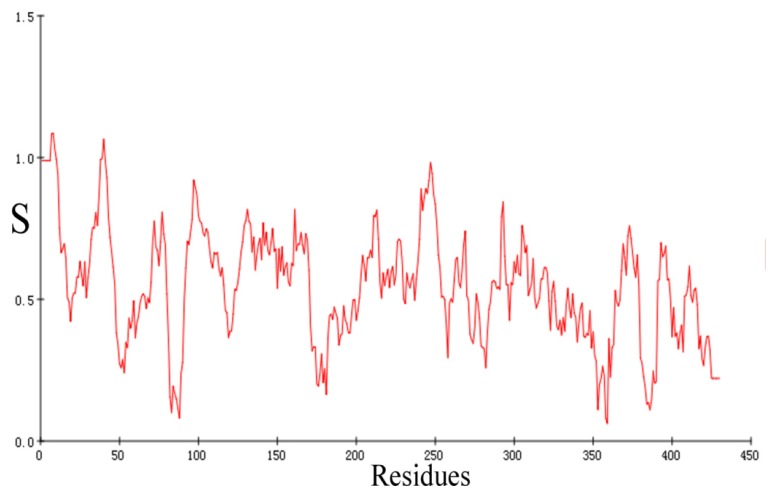
Verify-3D score of HSD.

Figure 4 represents the Verify 3D graph of the predicted HSD model. It is to be noted that compatibility scores above zero correspond to acceptable side chain environment. From Figure 4, we can see that almost all residues are reasonable. In brief, the geometric quality of the backbone conformation, the residue interaction, the residue contact and the energy profile of the structure is all well within the limits established for reliable structures. All evaluations suggest that a reasonable homology model for HSD has been obtained that can be exposed for examination of protein-substrate and protein inhibitor interactions.

### 2.3. Identification of Substrate-Binding Region and Co-Factor-Binding Region in HSD

HSDs have a common co-factor, NADH. It was reported that the NADH is bound to the Rossmann fold in the conventional mode, that is, the cofactor–enzyme interactions are predominantly mediated through hydrogen bonds between cofactor phosphate moieties and sugar hydroxyl groups with enzyme amide backbone groups [4]. However, the orientation of NAD^+^ of the nicotinamide ring is consistent with the experimentally observed stereospecificity of hydride transfer, that is to say that NADH hydride is facing the substrate-binding region.

The cavity volume estimated by CASTp [26] is dependent on the radius of the probe sphere; a probe radius of 1.4 Å outlines a cavity of 1124.2 Å^3^ for HSD of substrate binding, while a probe radius of 1.4 Å outlines a cavity of 1367.2 Å^3^ for 3MJT of substrate binding. From this result, we can conjecture that the active site of HSD is almost the same size of 3MJT.

Compared with the residues in the NADH binding site of the other HSDs’, the residues of HSD participating in the NADH binding site are listed in Table 1. In order to confirm whether the binding site determination for NADH is correct, the residues in the NADH-binding site of homoserine dehydrogenase (PDB ID 1EBF) [4] were coordinated with NADH that have been determined by X-ray. Seen from Table 1, we can conclude that the corresponding residues in HSD are Thr313, Val18, Gly19, Gly45, Gly47, Ile13, Ile46, Gly16, Gly14, Asn17, Ala105, Asn106, and Arg111. Half of these residues are conserved, and the reason may lie in the low sequence identity (27%). Hence the different NAD^+^-binding site residue between 1EBF and HSD may affect the catalytic efficiency of the substrate of these two enzymes.

### 2.4. Docking Study

The substrate, Aspartate-4-semialdehyde (ASA), and NADH are docked to HSD with Autodock 4.2 [27,28] and AutoDock vina [29,30], respectively. The grid size for Autodock 4.2 is 56 × 56 × 56 Å, and grid size for AutoDock vina is 24 × 24 × 24 Å. The results are listed in Table 2.

Seen from Table 2, the docking score is −5.24 Kcal·mol^−1^ for Autodock 4.2, while for AutoDock vina the docking score is 4.50 Kcal·mol^−1^. The RMSD between the original and the docked of the 3D structure of ASA are also shown in Table 2 (27.10 Å for AutoDock vina, 25.20 Å for Autodock 4.2). These results showed the binding mode generated by Autodock 4.2 is more reasonable than that of AutoDock vina, and thus chosen for further study.

**Figure 5.**
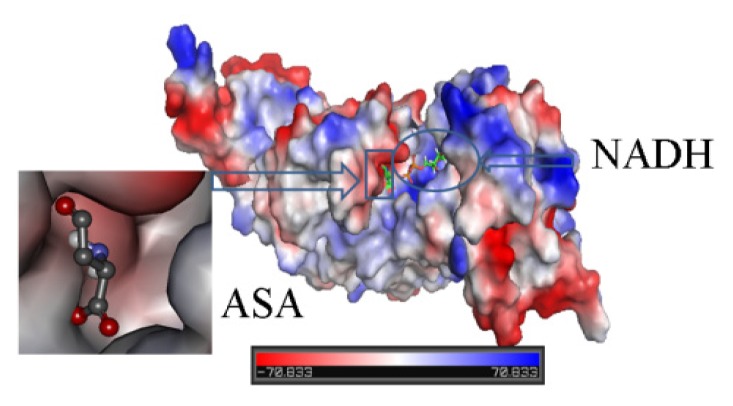
ASA and NADH bind in the HSD. They are located in different sites.

Figure 5 shows the substrate, ASA, and NADH docking in the HSD. We can see that ASA and NADH are in the groove. ASA is located near NADH, and so it is useful to transfer action.

**Figure 6.**
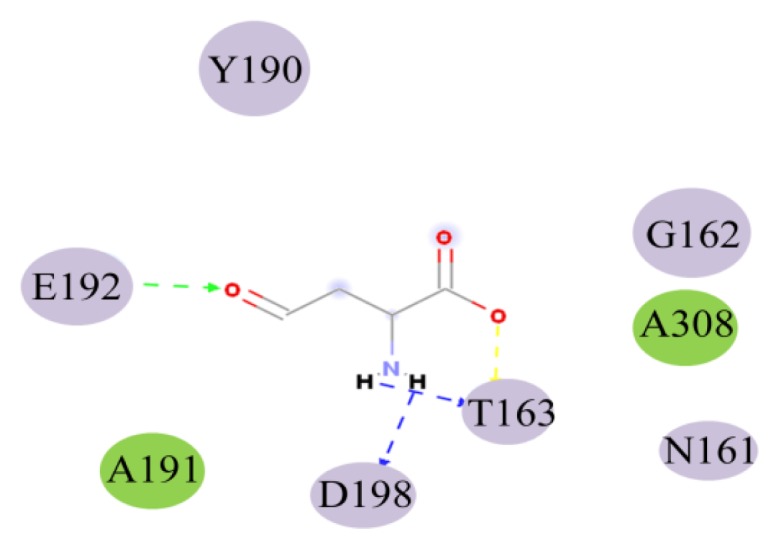
Active residues in the ASA binding pocket. The color purple represents strong van der Waals (VDW) contact with ASA, and the color green represents electronic contact with ASA.

Hydrogen bonds may be important in substrate binding. There are four hydrogen bonds between ASA and HSD (seen from Figure 6 and Table 3). Thr163 make two hydrogen bonds with the ASA (1.97 and 1.70 Å). Asp198 forms a strong hydrogen bon with the NH group with ASA. There is a weak hydrogen bond (2.26 Å) between the NH group of Glu192 and O8 atom of ASA. From Figure 6, it can be seen that Ala191 and Ala308 have electronic contact with ASA, whereas Tyr190, Glu192, Asp198, Gly162, Thr163, and Asn161 have strong van der Waals (VDW) contact with ASA. So the binding pocked of ASA to HSD may contain Ala191 and Ala308, Tyr190, Glu192, Asp198, Gly162, Thr163, and Asn161. In particular, Thr163, Asp198, and Glu192 may be important for ASA binding for they make hydrogen bonds with HSD. Glu192 is substrate binding residue, and this result is completely consistent with experimental data [15].

### 2.5. High Throughput Virtual Screening Procedure and Docking the Inhibitor to the Protein

**Figure 7.**
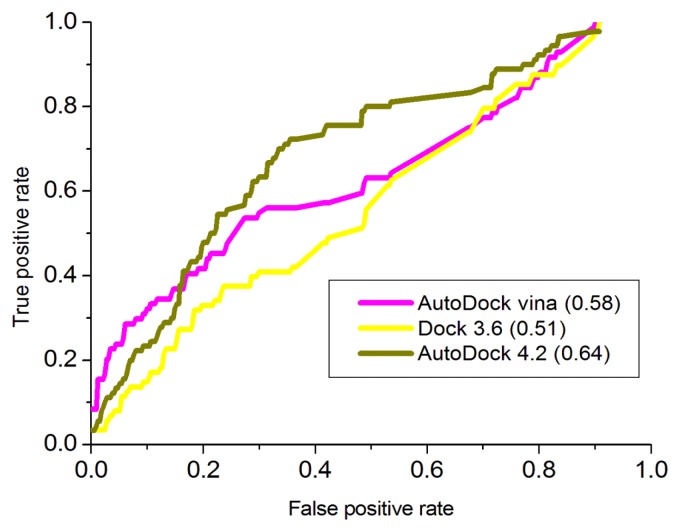
Receiving operating characteristic (ROC) curve.

It was reported that the receiving operating characteristic (ROC) evaluation is described as the ratio of the true positive rate to the false positive rate when a given proportion of known decoys have been observed [31,32]. Moreover, the receiving operating characteristic (ROC) curve, a graphical plot of the sensitivity (true positive rate, sensitivity) VS specificity (false positive rate), was calculated to avoid the sensitivity for small changes in ranking, where T score represents the number of correctly identified actives, and F score (false positives) represents the number of decoys incorrectly predicted as actives. The area under the ROC curve (AUC plot) gives the probability of ranking a randomly selected active higher than a randomly chosen decoy [33]. It ranges from 0 to 1, where 1 indicates a perfect ranking, where in all actives ranked above the decoys. A traditional academic point system for classifying the accuracy of a virtual screening test is known as follows: AUC < 0.5 is fail; 0.5 ≤ AUC < 0.70 is poor; 0.7 ≤ AUC < 0.8 is fair; 0.8 ≤ AUC < 0.9 is good; and 0.9 ≤ AUC ≤ 1 is excellent [34]. Essentially independent of the actual number of positive and negative instances, the AUC of an ROC plot gives an objective measure of query performance. In this study, 32 inhibitors [14,15,35,36] were used to generate the decoys using DUD-E on line [31]. Autodock 4.2 [27,29], Autodock vina [29,30] and Dock 3.6 [37] are used for docking. The ROC curve is shown in Figure 7. From Figure 7, AUC plot with Autodock 4.2 is 0.64, which is larger than that of AutoDock vina and Dock 3.6. And so Autodock 4.2 is used to further virtual screening.

Virtual screening of compound libraries has become a standard technology in modern drug discovery pipelines. The “2008/5” version of a Natural Products Database (NPD) contains almost 90,000 commercially available compounds. The target used in our study was the 3D structure of HSD mentioned above. In this simulation, it was also been screened that the potent inhibitor of HSD, 4-(4-HYDROXY-3-ISOPROPYLPHENYLTHIO)-2-ISOPROPYLPHENOL (178), which is found to be competitive with ASA (*IC*_50_ 5.1 μm) [15], as the leader drugs searching in Zinc data for 50% similarity.

After the screening, 164 compounds have been found. AutoDock 4.2 is used for virtual screening. AutoDock 4.2 uses a semi-empirical free energy force field to evaluate conformations during docking simulations. The force field was parameterized using a large number of protein-inhibitor complexes for which both structure and inhibition constants, or *K*_i_ are known. Table 4 listed the discovered inhibitors from the docking screen against the known crystal structure [14,15]. Two inhibitors showed good inhibition with the substrate ASA [14,15]. This result was consistent with our docking results (the free energy of binding and the calculated *K*_i_) (Table 4).

**Figure 8.**
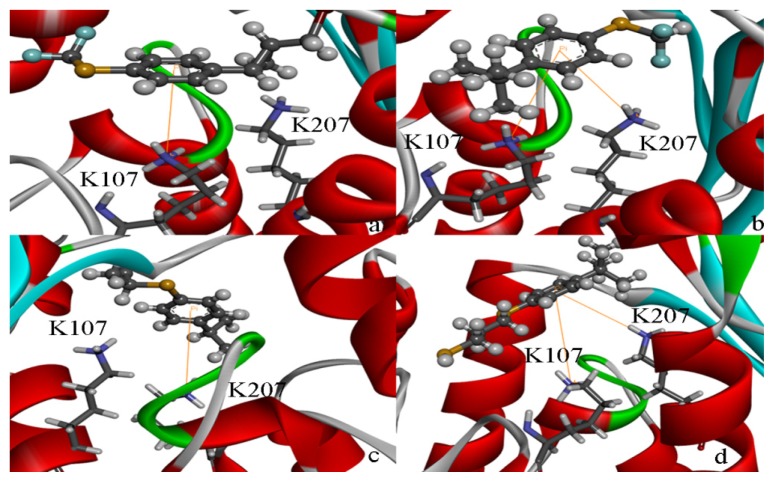
Predicted binding modes of ligands found from the homology model screen. (**a**–**d**) Predicted binding poses for four ligands discovered in the docking screen against the HSD homology model.

Figure 8 shows the binding pose of the inhibitor 178 in the HSD. In particular, Lys107 has a cation–π interaction with the inhibitor 178. The flat face of an aromatic ring has a partial negative charge owing to the pi electrons. Cations such as the sidechains of Lys or Arg, cationic ligands, or metal cations often align themselves centered over the faces of aromatic rings. It was reported that cation–π interactions should be considered alongside the more conventional hydrogen bonds, salt bridges, and hydrophobic effects in any analysis of protein structure, and they can also contribute significantly to intermolecular contacts and interactions with ligands [38–40]. As discussed before, the cation–π interaction makes the inhibitor-enzyme stable and cannot be removed.

**Figure 9.**
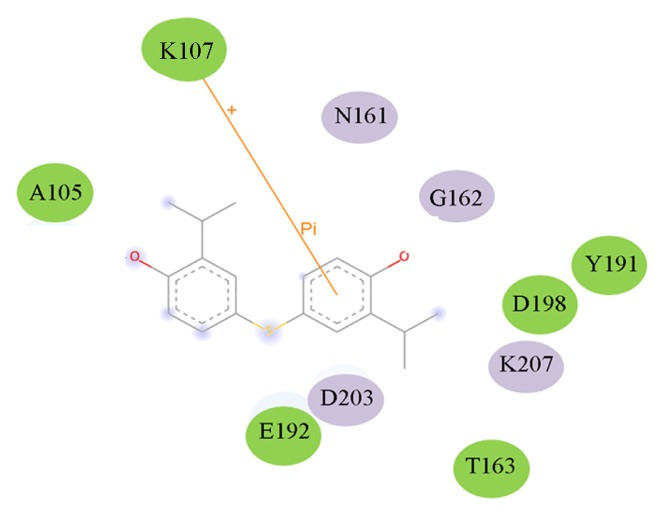
Active residues in the inhibitor 178 binding pocket. The color purple represents strong van der Waals (VDW) contact with178, and the color green represents electronic contact with178.

As shown in the Figure 9, in the HSD-178 complex, Ala105, Lys107, Glu192, Thr163, Asp198, and Tyr191 have electronic contact with inhibitor 178, and Asn161, Gly162, K207, and Asp203 have strong VDW contacts with inhibitor 178. In particularly, Lys107 forms a cation–π interaction with the inhibitor 178, and this result indicates Lys107 is an important residue for inhibitor 178. These results can serve as a guide to the selection of candidate sites for further experimental studies of site directed mutagenesis.

Table 5 lists four compounds. The binding energies and calculated *K*_i_ between four compounds and HSD are all lower than that of 178-HSD’s (−6.07 Kcal·mol^−1^, 17.56 μM). The similarity of the new compounds *versus* 178 was assessed by calculating the Tanimoto coefficient (*T*_c_) with Discovery studio 3.5 client to the 164 HSD inhibitors annotations. *T*_c_ value ranges from 0 to 1, where 0 represents no detection of the same bits; however, 1 does not mean that the two molecules are totally identical. The atom pair similarities (Sim_AB_) will be determined by the number of atom pair types shared by the two molecules, where 0 indicates no similarity and 1 indicates identity [32]. The four top-scoring docking hits (*T*_c_ < 0.6) is selected. The four top-scoring docking hits all seemed to cation–π ion pair with the key recognition residue Lys107, and Lys207 (from Figure 9). These ligands therefore seemed to be new chemotypes for HSD.

## 3. Experimental Section

### 3.1. Molecular Modeling

The amino acid sequence of the target protein, HSD, was obtained from UniProtKB/Swiss-Prot (No. P46806.1) and 441 residues were involved [18]. The template protein was homoserine dehydrogenase from *Thiobacillus denitrificans* (PDB Id 3MTJ sequence identifies 40%). The BLAST search algorithm was used for the online search [41]. Swiss Model [16,17] was employed to build the 3D structure. The modeling was then carried out using the Gromacs 4.3.5 software [42] with AMBER-03 all-atom force field. The temperatures were kept constantly at *T* = 300 K by coupling to a Berendsen thermostat with a coupling time of *T* = 0.1 ps. The protein was solvated using a box of TIP3P [43] water molecules extending at least 8 Å away from the boundary of any protein atoms. An integration step of 2 fs was used. Non-bonded interactions were calculated by using a cutoff of 8 Å. Long-range electrostatic interactions were calculated by Particle–Mesh Ewald summation with grid spacing of 1.2 Å and cubic interpolation. After 1000 steps of steepest descent energy minimization, the solvent and ions were equilibrated by 0.5 ns MD simulation with the protein heavy atoms subjected to harmonically constraints under a force constant of *k* = 1000 Kcal mol^−1^.nm^−2^. Finally, the production run was carried out for 20 ns, storing the coordinates of all the atoms each picosecond for further analysis.

### 3.2. Assessment of the Homology Model

To obtain an accurate homology model, it is very important that appropriate steps are built into the process to assess the quality of the model. Therefore, in the modeling phase, the model quality was assessed by the geometric quality of the backbone conformation, the residue interaction, the residue contact and the energy profile of the structure using different methods, including ERRAT [23] Verify 3D [24,25], and Molprobity [20–22].

### 3.3. Binding Pocket Analyses

The volume of the binding pocket is computed using the CASTp server [26,44] with default settings.

### 3.4. Validation of the Model by Docking Analysis

A docking study was conducted to evaluate the predictive ability of the HSD homology model and its suitability for use in the structure-based drug design studies. The structures of ASA, substrates of HSD were built using Chemdraw (Cambridge softInc, Cambridge, MA, USA). After a preliminary energy minimization to discard high-energy intramolecular interactions, the overall geometry and the atomic charges were optimized using Gaussian03 [30,45] software with 6-311G* set. In the validation phase, AutoDock 4.2 [29,30], AutoDock vina [31,32], and Dock 3.6 [33] were used for performing docking. In the process of new drug discovery, the application of virtual screening can enrich active compounds, reduce the cost of drug screening, and increase the feasibility of drug screening. Therefore virtual screening technology has become an important approach for new drug discovery. As virtual screening and bioactivity screening possess different advantages, their combination can effectively promote new drug discovery. In the present study the application and the trend of removal of non-drug compounds, removal of false positive compounds, molecular docking, and molecular similarity in the process of drug discovery are introduced in order to obtain more benefit from virtual screening strategy for new drug discovery.

#### 3.4.1. AutoDock 4.2

AutoDock combines a rapid energy evaluation through precalculated grids of affinity potentials with a variety of search algorithms to find suitable binding positions for a ligand on a given protein [29,30]. When docking was performed, HSD was kept rigid, but all the torsional bonds in ligands were set free to perform flexible docking. Polar hydrogens were added using the hydrogens module in AutoDockTools (ADT) for HSD; after that Kollman united atom partial charges were assigned [30]. Docking of ligands to HSD was carried out using the empirical free energy function and the Lamarckian genetic algorithm, applying a standard protocol with an initial population of 300 randomly placed individuals. Results were clustered according to the 1.0 Å root-mean square deviation (RMSD) criterions. All torsion angles for each compound were considered flexible. The grid maps representing the proteins in the actual docking process were calculated with AutoGrid. The grids (one for each atom types in the ligand plus one for electrostatic interactions) were chosen to be sufficiently large to include not only active site but also significant portions of the surrounding surface.

The software AutoDock 4.2 [29,30] was then applied in the virtual screening. A Natural Products Databse (NPD) [46] of Zinc database was employed. The target used in our study was the 3D structure of HSD mentioned above. Modification and format conversion of compounds which were downloaded from NPD used Open Babel toolbox [47] and Raccoon [48] graphical user interface for AutoDock [29,30] with a special focus on large-scale virtual screening.

There are 22 atom types in the docking course. The Parameters are listed as follow: ga_num_evals = 25,000,000, ga_run = 2 and ga_run = 50.

#### 3.4.2. AutoDock Vina

AutoDock Vina is a new open-source program for drug discovery, molecular docking and virtual screening, offering multi-core capability, high performance and enhanced accuracy and ease of use [31,32]. For its input and output, Vina uses the same PDBQT molecular structure file format used by AutoDock. PDBQT files can be generated (interactively or in batch mode) and viewed using MGLTools.

#### 3.4.3. Dock 3.6

DOCK improves the algorithm’s ability to predict binding poses by adding new features like force-field scoring enhanced by solvation and receptor flexibility [33].

## 4. Conclusions

This paper describes how a reliable and reasonable 3D structure of HSD was built with homology modeling techniques, and molecular dynamics methods. In order to determine the important residues of the substrate (ASA) binding, we docked the ASA to the protein. Thr163, Asp198, and Glu192 may be important residues for ASA binding. By means of virtual screening technique, 164 novel compounds were found. The four top-scoring docking hits all seemed to cation–π ion pair with the key recognition residue Lys107, and Lys207. We hope that our results are helpful for the future research of inhibitor design of HSD.

## Figures and Tables

**Table 1.** The different binding residues between 1EBF and HSD.

1EBF	HSD
Thr344	Thr313
Val16	Val18
Asn92	
Gly17	
Ala39	Gly45
Ile98	
Ala41	Gly47
Ile11	Leu43
Glu40	Ile46
Gly12	Gly14
Gly14	Gly16
Val15	Asn17
Thr93	
Pro115	Ala105
Asn116	Asn106
Arg117	Arg107

**Table 2.** The docking score between ASA and HSD with Autodock 4.2 and Autodock vina.

ASA	Docking score (Kcal·mol^−1^)	RMSD
Autodock vina	−4.50	27.10
Autodock 4.2	−5.24	25.20

**Table 3.** The hydrogen bonds between ASA and HSD.

Name	Distance (Å)	Donor atom	Acceptor atom	Angle
THR163:HN-ASA:O5	1.97	HN	O5	138.44
ASA:H9-THR163:OG1	1.70	H9	OG1	113.34
ASA:H10-ASP198:OD1	1.95	H10	OD1	116.55
GLU192:HN-ASA:O8	2.26	HN	O8	135.69

**Table 4.** Discovered ligands from the docking screen against the known crystal structure.

Structure	Enzyme	*K_i_* (μM)	Autodock 4.2 (Kcal·mol^−1^)	Estimated inhibition constant, *K_i_* (μM)
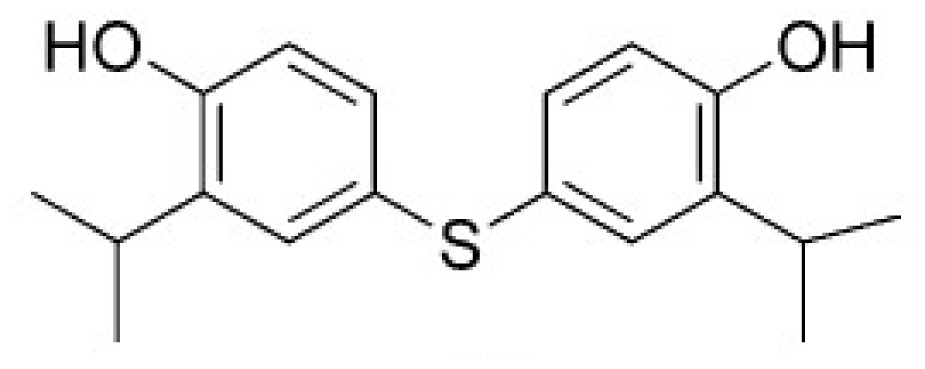	HSD (PDB ID 1TVE) [15]	10 ± 2	−8.15	9.92
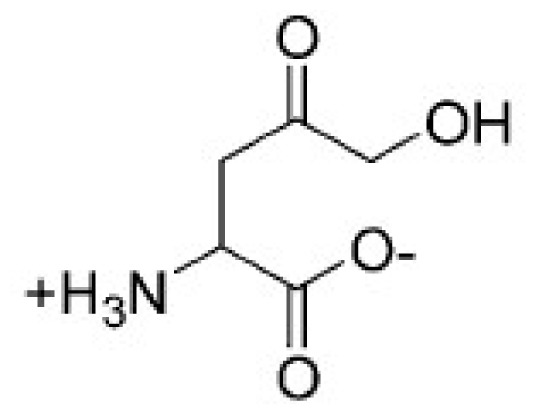	HSD (PDB ID 1Q7G) [14]	3.3 ± 0.9	−6.96	4.78

**Table 5.** The free binding energy among the inhibitors and HSD.

Compound	Structure	Autodock 4.2 (Kcal·mol^−1^)	*T*_c_^a^	*K_i_* (μM)
ZINC88161319	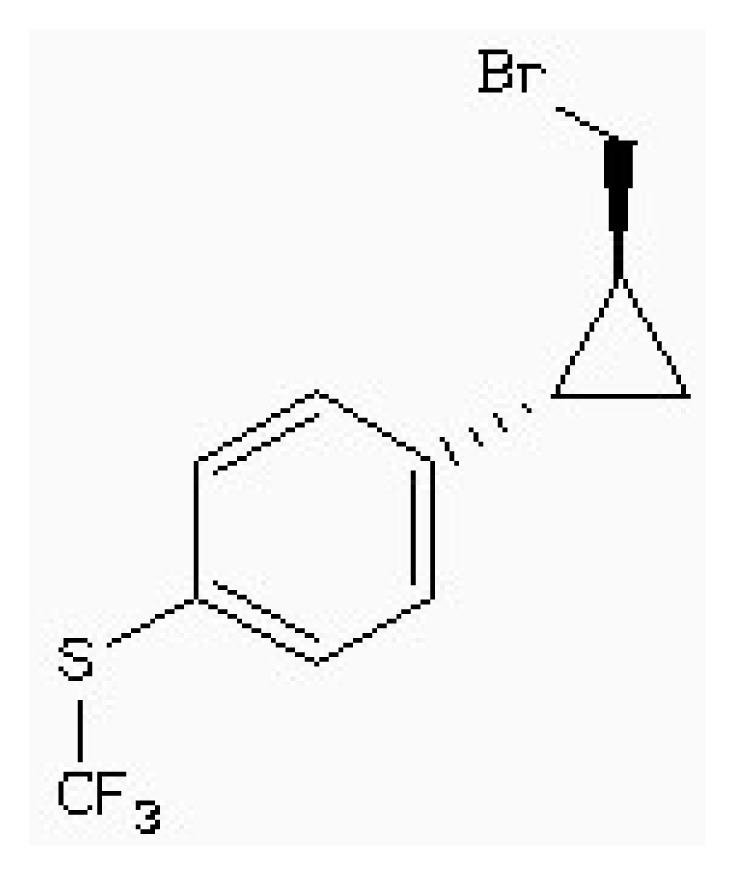	−7.28	0.52	9.63
Zinc41229093	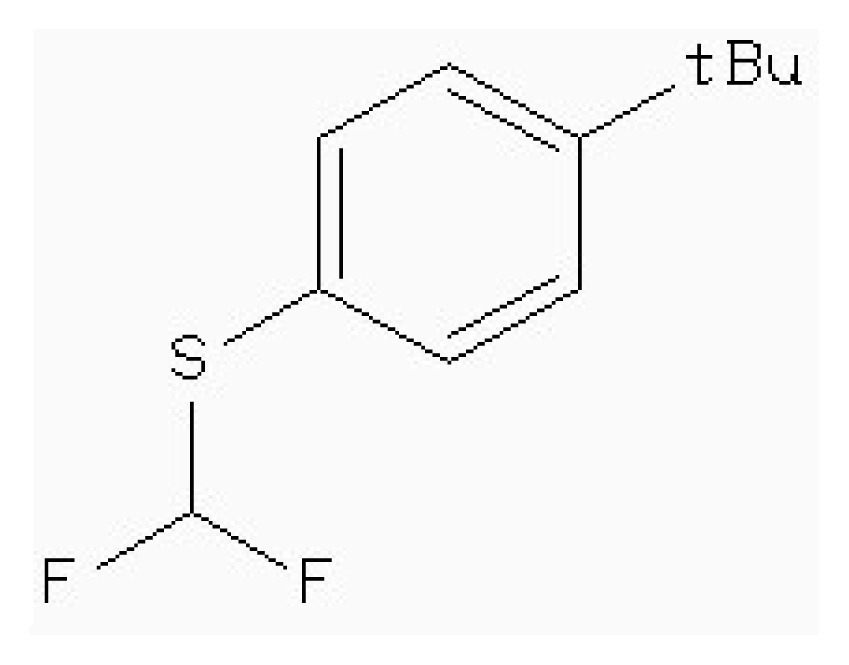	−7.09	0.55	13.07
Zinc54918273	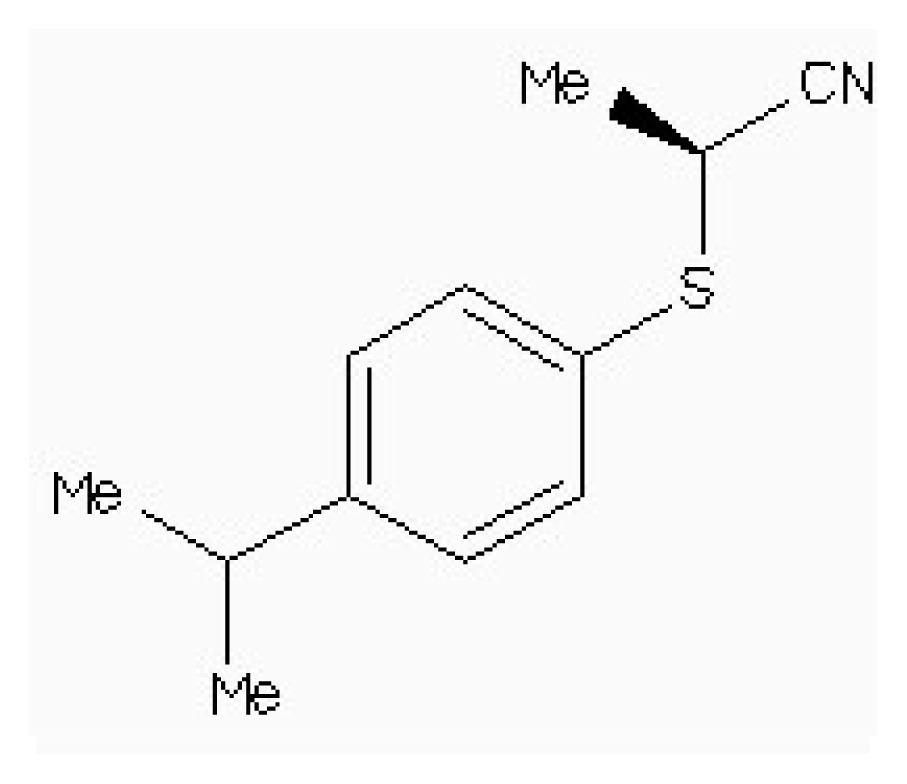	−6.66	0.40	13.10
Zinc87096659	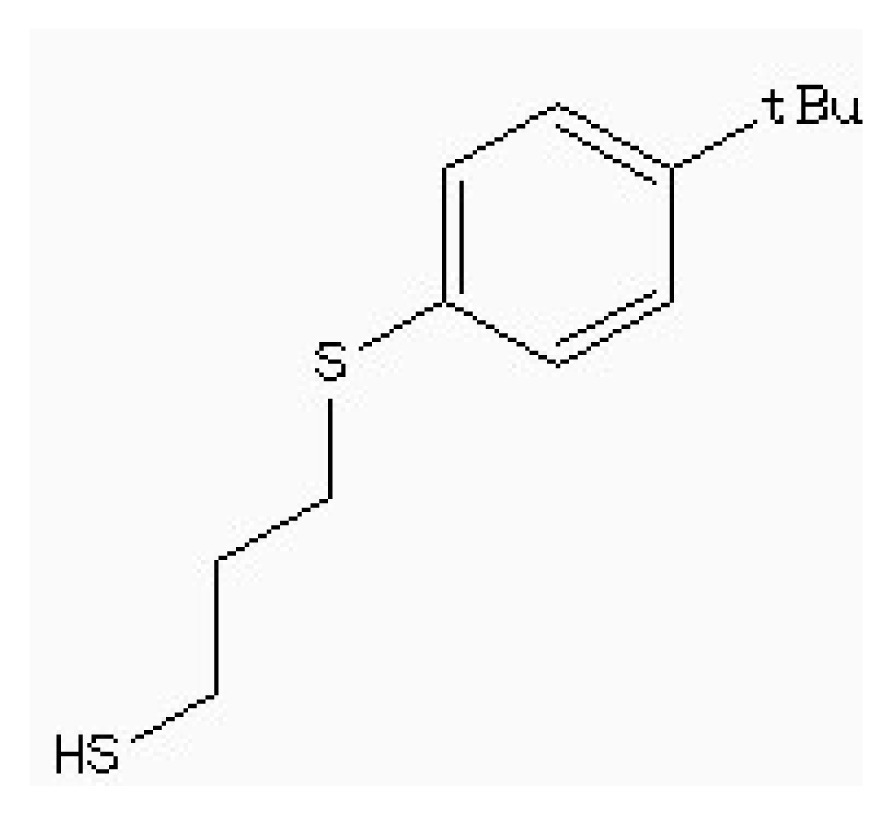	−6.48	0.38	15.68

a The tanimoto similarity (*T*_c_) to the most similar HSD inhibitor 178.
